# Tyrosinase-Expressing Neuronal Cell Line as *in Vitro* Model of Parkinson’s Disease

**DOI:** 10.3390/ijms11031082

**Published:** 2010-03-12

**Authors:** Takafumi Hasegawa

**Affiliations:** Department of Neurology Tohoku University School of Medicine, 1-1 Seiryomachi, Aobaku, Sendai, Miyagi 980-8574, Japan; E-Mail: hasegawa@em.neurol.med.tohoku.ac.jp; Tel.: +81-22-717-7189; Fax: +81-22-717-7189

**Keywords:** tyrosinase, Parkinson’s disease, cellular model

## Abstract

Oxidized metabolites of dopamine known as dopamine quinone derivatives are thought to play a pivotal role in the degeneration of nigrostriatal dopaminergic neurons in Parkinson’s disease. Although such quinone derivatives are usually produced *via* the autoxidation of catecholamines, tyrosinase, which is a key enzyme in melanin biosynthesis *via* the production of DOPA and subsequent molecules, can potentially accelerate the induction of catecholamine quinone derivatives by its oxidase activity. We have developed neuronal cell lines in which the expression of human tyrosinase was inducible. Overexpression of tyrosinase resulted in increased intracellular dopamine content in association with the formation of melanin pigments in neuronal somata, which eventually causes apoptotic cell death. This cellular model will provide a useful tool for detailed analyses of the neurotoxicity of oxidized catechol metabolites.

## 1. Introduction

Parkinson’s disease is a progressive neurodegenerative disorder characterized by the selective degeneration of dopamine (DA)-containing pigmented neurons mainly located in the substantia nigra. Neuromelanin-containing neurons are particularly susceptible to degeneration and their depigmentation is the hallmark of the advanced disease. The proposed mechanisms underlaying the pathogenesis and progression of neurodegeneration in the substantia nigra include iron-catalyzed oxidative stress, mitochondrial dysfunctions, inflammation and disturbances of protein metabolism [1–3]. DA itself can be autoxidized to form reactive species, DA quinone, which may be covalently incorporated into a variety of molecules including proteins, lipids, and nucleic acids [4–8]. In addition, highly reactive oxygen species (ROS; hydrogen peroxide, superoxide radical, and hydroxyl radical) are generated, not only in catecholamine oxidation, but also during the decay of redox-active quinones [9]. Several targets have been identified for these catechol quinones in neurons. In the cytosol, DA quinone can react with the sulfhydryl group of cysteine residues to form protein adducts, thus leading to irreversible alteration of protein function. DA oxidation inhibits DA transporter glutamate transporter, and mitochondrial respiratory chain function [10]. Furthermore, DA quinone can interact with α-synuclein, a protein responsible for familial Parkinson's disease [11], and form a toxic intermediate in nigral cells [12]. At physiological pH DA quinone cyclizes irreversibly and spontanenously to form dopaminochrome (also celled aminochrome) and it is a precursor of neuromelanin. Such one-electron reduction of DA quinone is potentially neurotoxic and it has been reported that aminochrome is the metabolite of DA oxidation involved in the formation of α-synuclein adducts inducing and stabilizing cytotoxic protofibrils [13,14]. These findings suggest that DA-derived reactive species may interact with some key molecules in neuronal cells, thereby leading to neuronal cell death and the concomitant neuropathological changes.

**Figure 1.**
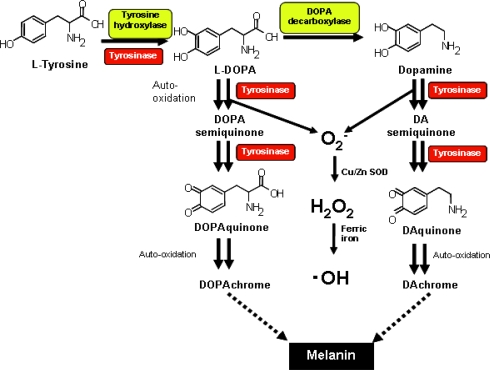
Generation of cytotoxic catechol *o*-quinones and other reactive oxygen species during the enzymatic action of tyrosinase.

Tyrosinase (EC 1.14.18.1; monophenol monooxygenase), a copper-containing metalloprotein, is a key enzyme in the biosynthesis of melanins and other polyphenolic compounds [15]. Tyrosinase catalyzes both the hydroxylation of tyrosine to l-DOPA and the subsequent oxidation of l-DOPA to dopaquinone (Figure 1) [16,17]. In addition, under certain circumstances tyrosinase may oxidize DA to form melanin pigments, probably through DA quinone [18,19]. Therefore, tyrosinase is the major enzyme required for the synthesis of melanin in skin and hair and may contribute to neuromelanin formation. In mammals, tyrosinase is specifically expressed in differentiated melanin-producing cells such as melanocytes that are distributed in the skin and retinal pigment epithelium [20]. In the mammalian central nervous system, a DA-derived pigment, neuromelanin, is found in nigral dopaminergic cells [21]. The existence of tyrosinase in the central nervous system is still controversial, although several studies have shown the expression of tyrosinase gene, its protein, and its catalytic activity in the nigral neurons [18,22–24]. Greggio *et al* has shown that tyrosinase activity is higher in substantia nigra, a region rich in DA, the major tyrosinase substrate.

To obtain better understanding of the potential toxicity of oxidized dopamine metabolites in pigmented neuronal cells, we established neuronal cell lines that express human tyrosinase in response to doxycycline, an exogenous inducer [25–28]. Tyrosinase induction leads to apoptotic cell death in concomitant with the increased production of intracellular DA and ROS, followed by the formation of pigmented granules mimicking those containing neuromelanin that are seen in nigral cells.

## 2. Establishment of Neuronal Cell Lines Inducible for the Expression of Human Tyrosinase

**Figure 2.**
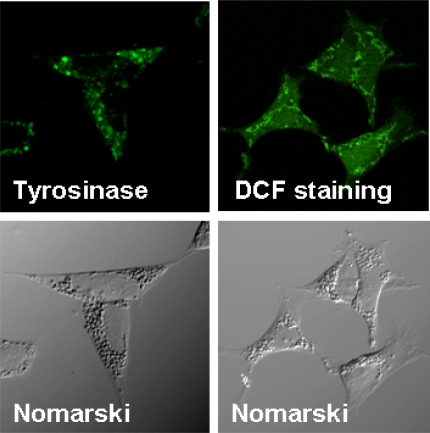
Inducible expression of human tyrosinase in SH-SY5Y cell. Immunohistochemical staining of SH-SY5Y cell line expressing tyrosinase under tetracycline regulatable promoter. At 72 hrs after the induction with doxycycline (1 g/mL), dispersed cytoplasmic tyrosinase protein (green) had accumulated in cytoplasmic particles, which were co-localized with rounded, pigmented bodies mimicking the neuromelanin granules. Intracellular ROS production was visualized by ROS indicator DCFH_2_DA (green fluorescence).

We used the T-REx tetracycline-regulatable gene expression system (Invitrogen, Carlsbad, CA, USA) to induce human tyrosinase in dopaminergic SH-SY5Y neuronal cells. To generate the expression construct of human tyrosinase (pcDNA4(TO)-tyrosinase), human tyrosinase cDNA was cloned at *Xho*I/*Xba*I sites in the mamalian expression vector pcDNA4(TO). Tet-repressor protein was encoded by the pcDNA6/TR regulatory vector. Stable SH-SY5Y neuroblastoma cell lines expressing human tyrosinase under the transcriptional control of the T-REx system were then cloned by successive expression of the hybrid Tet repressor and pcDNA4(TO)-tyrosiase under the existence of selection antibiotics. In the absence of doxycycline, the expression of tyrosinase was repressed by the binding of Tet-repressor homodimers to the tetracycline operator sites in the pcDNA4(TO)-tyrosinase. The addition of doxycycline (1 μg/mL) resulted in a derepression of promoter activity, leading to expression of the tyrosinase protein. Three days after the doxycycline induction, the tyrosinase protein as well as intracellular ROS had accumulated in cytoplasmic granules that exhibited cobblestone-like pigmented structures similar to the neuromelanin granules seen in nigral cells (Figure 2).

For proper melanogenesis in pigment cells, tyrosinase should be *N*-glycosylated in the endoplasmic reticulum (ER), modified further in the Golgi apparatus and then translocated into melanosomes [29,30]. During these processes, the interaction of tyrosinase with the ER chaperone calnexin is indispensable for the protein folding [31]. We confirmed that ER marker calnexin surrounded the assembled tyrosinase, suggesting the proper interaction of these two molecules. In addition, the tyrosinase-containing structures were co-stained with the lysosomal marker LysoTracker, suggesting that the expressed tyrosinase was targeted to lysosomes. This observation is consistent in part with the notion that both melanosome and neuromelanin are derived from specialized lysosomes [32–34] and that the targeting signals for melanosomes and lysosomes are functionally equivalent and exchangeable [35]. Thus, the expressed tyrosinase may be sorted to lysosomes in the established neuronal cell lines that lack melanosomes.

The expressed tyrosinase in SH-SY5Y cells showed the DOPA oxidase activity but also oxidase activity on DA, suggesting that the expressed tyrosinase could catalyze the oxidation of DA. The intracellular DA contents measured by HPLC-EC increased significantly after the overnight induction of tyrosinase. In parallel with the formation of cytoplasmic pigmented granules, significant increase of intracellular melanin, the oxidized products of DOPA and DA, was observed after the tyrosinase induction. Thus, intracellular DOPA produced by tyrosinase was not only oxidized to dopaquinone but also converted to DA by intrinsic dopa decarboxylase in catecholaminergic cells.

## 3. Overexpression of Tyrosinase Induces Neuronal Cell Death *via* Activation of SAPKs and Caspase 3 Apoptosis Pathway

**Figure 3.**
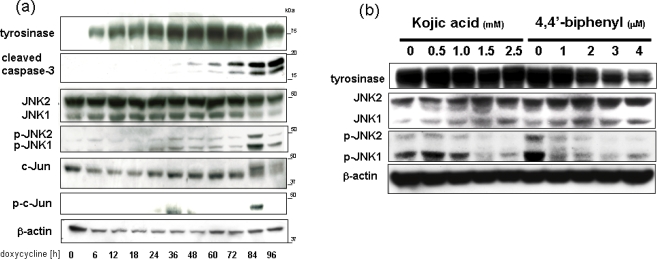
Tyrosinase induction causes activation of SAPK and caspase-3 pathways. (a) Tyrosinase expression was induced for the indicated times with doxycycline. Samples were subjected to Western blot analysis and sequentially probed for tyrosinase, procaspase-3 and the activated, cleaved form of caspase-3, JNKs, c-Jun and their activated, phosphorylated forms, as indicated. Reprobing the blots for □β-actin confirmed equal loading. (b) Kojic acid and 4,4’-dihydroxybiphenyl significantly block the tyrosinase-mediated JNK-c-Jun activation.

The oxidative stress generated by tyrosinase induction is likely to involve DA quinones, which are well known to be neurotoxic [10,24]. Indeed, tyrosinase induction in SH-SY5Y cells caused cell death, as measured by LDH release assay and MTT assay. This result is in good agreement with the previous reports showing that artificial expression of tyrosinase in non-pigmented cells triggered the cascade of melanin synthesis and produced cytotoxic intermediates followed by growth retardation and/or cell death [36–38]. Upon tyrosinase induction, cell death occurred in a time dependent manner. We focused on the caspase 3 cleavage and activation of the classical MAPK cascades since one major pathway of PD-relevant DA neurotoxicity involves the c-Jun N-terminal kinase (JNK). In a detailed time course experiment, doxycycline treatment augumented tyrosinase protein expression already after 6 h (Figure 3a). Tyrosinase levels continued to rise until a high level was reached between 36 and 72 h post-induction. Cleavage of the effector apoptosis caspase 3 consistent with caspase activation became first apparent after maximal induction of tyrosinase, and continually increased to the prolonged time points beyond 72 h. Involvement of the MAPK pathways in tyrosinase-induced apoptotic signaling was determined using phosphor-specific antibodies recognizing activated forms of MAPKs. Basal phosphorylation of p44/p42 MAPK was not changed upon tyrosinase induction. In contrast, activation of the JNK-c-Jun pathway became detectable after 36 h. The time course of SAPK stimulation was similar to caspase 3 cleavage and LDH release. The tyrosinase inhibitor kojic acid and 4,4’-dihydroxybiphenyl effectively block the JNK-c-Jun activation and subsequent neuronal cell death, suggesting that the induction of tyrosinase enzyme specifically causes neurotoxicity in SH-SY5Y cells (Figure 3b).

## 4. Conclusions

We have established catecholaminergic neuronal cell lines that express tyrosinase in response to doxycycline, an exogeneous inducer. In these cell lines, the overexpressed tyrosinase protein increases the content of DA and ROS, followed by the production of melanin pigments, and finally leads to apoptotic cell death. Although catechol oxidized metabolites are likely to be toxic, neuromelanin formation itself could be protective by converting cytotoxic excess DA to stable neuromelanin. In contradiction to our results, it was previously demonstrated that the inhibition of tyrosinase reduced cell viability and in catecholaminergic CATH.a cell. This disagreement would be attributed to the different types of cells in each study. SH-SY5Y cells used in our study express quite lower level of DA at stable state than DA-rich primary mesencephalic neuron or CATH.a cell, indicating that SH-SY5Y cell would be more vulnerable against catechol metabolites because of its poor anti-oxidative system.

It is still controversial whether tyrosinase is involved in the neuromelanin-biosynthetic pathway, however, as a model system, this cellular model provides a useful tool for exploring the possible roles of catecholamine and its oxidized metabolites or certain environmental factors in the pathogenesis of Parkinson’s disease and related neurodegenerative disorders.

## Abbreviations:

DA: dopamine
ROS: reactive oxygen species
TH: tyrosine hydroxylase
ER: endoplasmic reticulum
DCF: 2,7-dichlorofluorescein
SAPK: stress-activated protein kinase
MAPK: mitogen-activated protein kinase
JNK: c-Jun *N*-terminal kinase
